# Convolutional Neural Networks Using Enhanced Radiographs for Real-Time Detection of *Sitophilus zeamais* in Maize Grain

**DOI:** 10.3390/foods10040879

**Published:** 2021-04-16

**Authors:** Clíssia Barboza da Silva, Alysson Alexander Naves Silva, Geovanny Barroso, Pedro Takao Yamamoto, Valter Arthur, Claudio Fabiano Motta Toledo, Thiago de Araújo Mastrangelo

**Affiliations:** 1 Center for Nuclear Energy in Agriculture, University of São Paulo, Piracicaba 13416-000, SP, Brazil; arthur@cena.usp.br (V.A.); piaui@cena.usp.br (T.d.A.M.); 2 Institute of Mathematics and Computer Sciences, University of São Paulo, São Carlos 13560-970, SP, Brazil; naves@usp.br (A.A.N.S.); claudio@icmc.usp.br (C.F.M.T.); 3 Department of Entomology and Acarology, College of Agriculture Luiz de Queiroz, University of São Paulo, Piracicaba 13418-900, SP, Brazil; geovannybarroso@usp.br (G.B.); pedro.yamamoto@usp.br (P.T.Y.)

**Keywords:** deep learning architectures, MobileNetV2, Xception, Inception-ResNet-v2, peripheral equalization, calcification emphasis, transfer learning, ImageNet, X-ray imaging, maize weevil

## Abstract

The application of artificial intelligence (AI) such as deep learning in the quality control of grains has the potential to assist analysts in decision making and improving procedures. Advanced technologies based on X-ray imaging provide markedly easier ways to control insect infestation of stored products, regardless of whether the quality features are visible on the surface of the grains. Here, we applied contrast enhancement algorithms based on peripheral equalization and calcification emphasis on X-ray images to improve the detection of *Sitophilus zeamais* in maize grains. In addition, we proposed an approach based on convolutional neural networks (CNNs) to identity non-infested and infested classes using three different architectures; (i) Inception-ResNet-v2, (ii) Xception and (iii) MobileNetV2. In general, the prediction models developed based on the MobileNetV2 and Xception architectures achieved higher accuracy (≥0.88) in identifying non-infested grains and grains infested by maize weevil, with a correct classification from 0.78 to 1.00 for validation and test sets. Hence, the proposed approach using enhanced radiographs has the potential to provide precise control of *Sitophilus zeamais* for safe human consumption of maize grains. The proposed method can automatically recognize food contaminated with hidden storage pests without manual features, which makes it more reliable for grain inspection.

## 1. Introduction

*Sitophilus zeamais* Motschulsky (Coleoptera: Curculionidae) is one of the most serious pests of stored maize grain worldwide, particularly in tropical and sub-tropical regions [1,2], causing huge economic losses in agricultural and food industry. This weevil also attacks several other cereals and agricultural products, processed or not [3]. In maize grain, the *Sitophilus zeamais* life cycle is 150 days [4]. Hence, the damage caused by feeding of larvae, pupae and adults can significantly reduce the weight and quality of the grains during storage, which facilitates the entry of pathogens and mites [5].

In many countries, there is an increasing trend towards zero-tolerance for stored-grain insects [6]. However, the early detection of *Sitophilus zeamais* in grains is very difficult to be achieved in practice because the egg, larva and pupa development occur inside the grain, which is not perceived by the human eye. Different methods have been developed to identify signs of insect infestation during storage such as staining of grains, acoustic techniques, Berlese funnel, uric acid method, grain probes and insect traps [6,7]; however, these methods are time-consuming and require experienced technicians, and their accuracy depends on the insect development stage and infestation level. In fact, most conventional techniques are useful only for external detection [7].

The development of advanced methods for the early detection of grain pests is essential in the food industry that can help in decision-making. Soft X-ray imaging is a fast, non-destructive and accurate technique for internal and external detection of insects in stored food grains [8], regardless of the life stage of the insect. Furthermore, recent algorithms focused on X-ray image contrast enhancement, including peripheral equalization and calcification emphasis enable superior diagnostic images. These algorithms provide the opportunity to distinguish finer density differences between features in the image [9,10]. Nevertheless, the automatic inspection of insect infestation is still a challenge. For instance, limited research has attempted to use image processing algorithms to identify infested grains. In addition, the application of mathematical and computational methodologies requires interdisciplinary knowledge.

Recently, deep learning methods have been applied to solve many classification problems from robotics, games and medicine [11]. Convolutional neural networks (CNNs) are considered the dominant deep learning models [12], with strong potential in performing classification tasks [13]. This technique uses artificial neural network (ANN) architectures, which attempt to mimic how neurons work and interact with the world [14]. Several “neurons” (multiple layers) act in concert as parallel information processors to automatically recognize patterns in data with high precision [15]. The growing interest in applying CNNs architectures has been mainly due to two factors: (i) the large number of databases and (ii) the advancement in hardware, which reduces the processing time of these databases [16].

CNN architectures have been particularly used for image detection, segmentation and classification because images have a special spatial property in their formation, such as edges, textures, gradients, orientation and color [15]. Many deep learning architectures have been proposed for automatic pattern recognition, such as the Inception-ResNet-v2, Inception-v3, VGG19, ResNet-50, DenseNet-201, Xception and MobileNetV2 architectures, with different performances depending on the characteristics of the data [17,18,19,20,21,22,23]. These CNN architectures have enabled the development of human-like efficient machines in different domains of application [15].

Considering the great potential of deep learning models, they are ideal candidates to provide predictions and recommendations during the monitoring of stored products. Here, we propose the application of radiography using CNNs models to discriminate classes of non-infested maize grains and grains infested with *Sitophilus zeamais*. Our study included the application of image processing techniques based on peripheral equalization and calcification emphasis algorithms to improve the detection of *Sitophilus zeamais* in the initial infestation phase, in which the damages are more difficult to detect. We explored three different CNN-based neural architectures widely used for classification tasks, (i) Inception-ResNet-v2, (ii) Xception and (iii) MobileNetV2, which allowed to learn complex prediction models. To the best of our knowledge, this is the first attempt to use peripheral equalization and calcification emphasis algorithms for X-ray images of food products combined with deep learning approaches.

## 2. Materials and Methods

### 2.1. Insect Infestation

Ten sets of twenty pairs of *Sitophilus zeamais* adults (1 male: 1 female) were allowed to mate in plastic containers (300 mL) containing 20 g of maize grains. Five containers with 20 g of non-infested grains represented the control. The containers were covered with a perforated lid and kept at 25 ± 2 °C and 65 ± 10% relative humidity with a photoperiod of 14/10 h of light/dark. After 24 h, the males were removed and the females remained isolated to lay eggs during four days. Then, the females were removed from the containers and the insect infestation was monitored every seven days for 42 days. 

### 2.2. X-ray Imaging for Classes of Infested and Non-Infested Grains

Radiographic images were acquired from all samples at 7, 14, 21, 28, 35 and 42 days using a MultiFocus™ instrument (Faxitron Bioptics LLC, Tucson, AZ, USA). This system is integrated with a complementary metal-oxide-semiconductor (CMOS) X-ray sensor coupled with an 11 μm focal spot tube, providing high-resolution grayscale images of 3072 × 2148 pixels (6 μm/pixel). Initially, the exposure time of the samples and the voltage settings were automatically set by the sensor. Based on the pre-established configurations, the exposure time of 6.0 s and 27 kV was manually standardized. To emphasize the smaller details in the image, we used a built-in function in MultiFocus™ software (Faxitron Vision NDT version 2.3.2U B) named “enhance image”. This image processing technique uses peripheral equalization and calcification emphasis algorithms [24,25]. These algorithms are widely used to detect abnormalities in mammograms such as calcification, masses and architectural distortion that are too subtle to be perceived clearly by radiologists. Therefore, this tool was used to enable superior diagnostic radiographic images, particularly in the egg stage and first instar larva in which the damages are more difficult to detect.

Red-green-blue (RGB) images were also acquired using SeedReporter™ equipment (PhenoVation B.V., Wageningen, The Netherlands), generating high-resolution images with a spatial dimension of 2448 × 2448 pixels (3.69 μm/pixel). The RGB images are represented by three-color channels (red, green and blue) to generate a single-color value for each pixel in the image, and they were captured to assess possible symptoms on the surface of the grains.

### 2.3. Confirmation of Grains Infested by Eggs

To confirm that the symptoms shown in the radiographic images were related to the presence of eggs, a second experiment was carried out following the same procedures as those adopted in Section 2.1. After X-ray imaging, the grains were stained with 0.5% acid fuchsin solution (0.5 g acid fuchsin + 50 mL glacial acetic acid + 950 mL distilled water) for 3 min [26]. Subsequently, each grain was examined individually using a stereomicroscope to locate the egg plugs.

### 2.4. Datasets

The major challenges for deep learning projects are the lack of reliable data. The success of the training is closely linked to the number of varied data that were collected. Since there is a lack of pre-existing databases, a dataset with high-quality images was built with 426 radiographic images separated into two classes: (i) 270 infested grains and (ii) 156 non-infested grains. The datasets comprised only X-ray images processed by the peripheral equalization and calcification emphasis algorithms.

Later, data were separated into training (70% of the images) and validation sets (30% of the images). The training set comprised 181 images from infested class and 117 images from non-infested class, divided into 19 training batches (18 batches with 16 images and 1 batch with 10 images). The validation set contained 89 images from the infested class and 39 images from the non-infested class, divided into 8 batches with 16 images each. One of the batches in the validation set was transformed into a test set, containing 16 images. To build the predictive models, all images were resized to 160 × 160 pixels for training, validation and test sets.

### 2.5. Data Augmentation

CNNs have many weights that must be defined, and they therefore require a large number of labeled data to learn accurately [16]. To ensure variation in the training dataset, increasing the volume of data, and transforming the invariant models to image features that do not affect their labels, data augmentation methods can be used. We applied data augmentation only to the training set, creating more images. The images were pre-processed by geometric transformations based on rotations and flips (mirror effect) that allow the repositioning of the pixels in an image while maintaining the neighborhood relationship between them and preserving the visual characteristics of the image.

**Figure 1:**
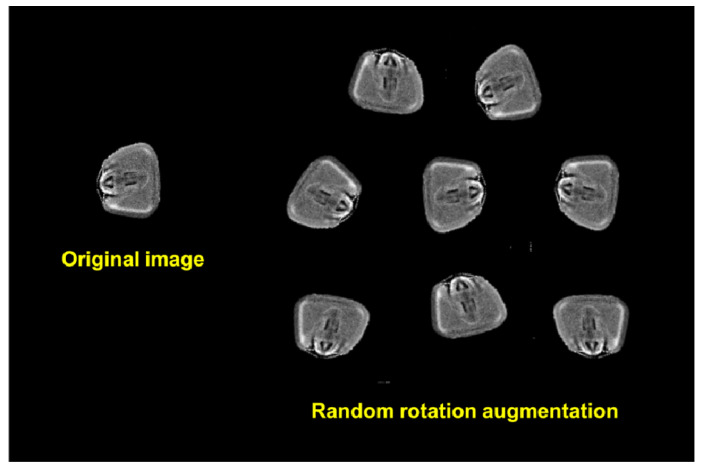
Data augmentation method based on random rotations and flips (mirror effect) used for X-ray image recognition in classes of maize grains infested and non-infested by *Sitophilus zeamais*.

The images were rotated clockwise or counterclockwise, with angles randomly defined (Figure 1). This method uses bilinear interpolation that preserves straight-line features through the image, producing a smoother interpolation than does the nearest neighbor approach. The flip transformation combines image rotation by multiple angles of 90° with the calculation of the transposed matrix of the original image pixel, reversing the pixels horizontally or vertically. A horizontal flip rotates the image 90° clockwise or 270° counterclockwise under transposition of the original image, while a vertical flip rotates the image 90° counterclockwise or 270° clockwise under the transposed matrix.

### 2.6. Transfer Learning and Architecture Approaches

Transfer learning consists of applying knowledge previously acquired by a certain domain, adapting it to solve a new problem in a different context of images [27]. There are two common methods for transfer learning approaches. The first is performed by freezing the weights from lower convolutional layers that were adjusted and learned from another database and only training the dense layer according to the target classes [28]. In this case, transfer learning facilitates the training process because it uses the feature extractor from other tasks, avoiding the weight adjustment in the convolutional layers. In addition, training adjusts the weights of the upper classification layers in the deep network, which is useful when we do not have a large amount of data to train all the weights. In the second method, a fine tuning based on the lower layer weights is used as the initial point and the weights of all (or some) layers of the network are refined. We adopted the first strategy.

**Figure 2:**
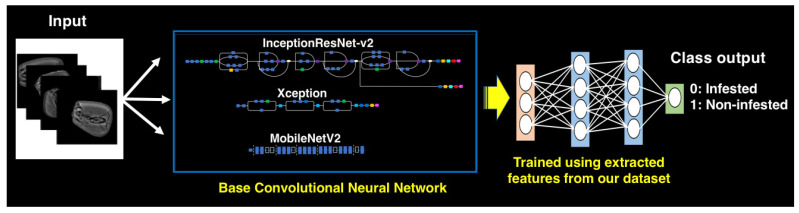
Outline of the convolutional neural network models developed for X-ray image classification of maize grains infested and non-infested with *Sitophilus zeamais* using the Inception-ResNet-v2, Xception and MobileNetV2 architectures.

We compared the performance of three different architectures, (i) Inception-ResNet-v2 [29], (ii) Xception [30] and (iii) MobileNetV2 [31] (Figure 2), and the weights of the convolutional layers were trained on the ImageNet dataset [32]. We used models with pre-trained weights of the convolutional layers, and the last fully connected layer was replaced with a single neuron in the output layer with loss function binary cross-entropy. The output neuron contains probabilities (0 ≤ *p* ≤ 1) that the input image belongs to class 0 (infested) or 1 (non-infested).

Table 1 shows the proposed CNNs for X-ray image classification of maize grains infested and non-infested by *Sitophilus zemais* with their parameters and accuracies in the ImageNet dataset. The size refers to the file size with pre-trained weights. The top-1 and top-5 accuracies refer to the performance of the models on the ImageNet validation dataset, where top-1 represents the class with the highest probability to match the target label, and top-5 accuracy represents the five predictions of the model with the highest probability to match the target label. Depth refers to how deep the CNN is, including the convolutional layers, activation and batch normalization. The number of trainable parameters corresponds to the number of weights that must be trained with our dataset, while the number of non-trainable parameters is related to the number of pre-trained weights in the ImageNet dataset. We used Adam (Adaptive Moment Estimation) optimization to minimize the error rate of the model in the prediction [33], which is a stochastic gradient descent method based on adaptive estimation of first and second-order moments, with a learning rate equivalent to 10^−4^ and the binary cross-entropy loss function that calculates the cross-entropy loss between the actual and predicted labels.

All models were trained for 200 epochs in the Google Colaboratory, also known as Google Colab or Colab, which is a free environment used to implement Python algorithms in a Jupyter Notebook interface with access to Google hardware. Linux-based virtual machines (VMs) are provided by Google, where Notebooks can be processed in central processing units (CPUs), graphics processing units (GPUs) or in tensor processing units (TPUs). The hardware available to the user varies by session. The training was processed with an Intel (R) Xeon (R) CPU @ 2.20 GHz, 13 GB RAM, NVidia Tesla T4 and GDDR6 16 GB VRAM. The experiments were developed using Python, Keras and Tensorflow, which are libraries focused on machine learning.

### 2.7. Confusion Matrix and Metrics

Confusion matrices were created for the developed models, which reveal the number of correct and incorrect predictions for each class in a given dataset. The performance of the models was evaluated using five metrics—precision, accuracy, sensitivity (recall), specificity and F1 Score (harmonic mean of precision and sensitivity) [34,35]—according to the following formulas:

(1) $$\;\;\;\;\mathrm{Precision}=\frac{\mathrm{TP}}{\mathrm{TP}+\mathrm{FP}}$$

(2) $$\mathrm{Accuracy}=\frac{\mathrm{TP}+\mathrm{TN}}{\mathrm{TP}+\mathrm{TN}+\mathrm{FP}+\mathrm{FN}}$$

(3) $$\mathrm{Sensitivity}=\frac{\mathrm{TP}}{\mathrm{TP}+\mathrm{FN}}$$

(4) $$\mathrm{Specificity}=\frac{\mathrm{TN}}{\mathrm{TN}+\mathrm{FP}}$$

(5) $$F1\;\mathrm{Score}=\frac{2\mathrm{TP}}{2\mathrm{TP}+\mathrm{FP}+\mathrm{FN}}$$

TP (True Positive) represents the number of X-ray images from infested grains correctly classified as infested grains, TN (True Negative) is the opposite, i.e., X-ray images from non-infested grains correctly classified as non-infested grains, FP (False Positive) refers to non-infested grains incorrectly classified as infested grains and FN (False Negative) represents infested grains incorrectly classified as non-infested grains.

## 3. Results

### 3.1. Classes of Infested and Non-Infested Grains

**Figure 3:**
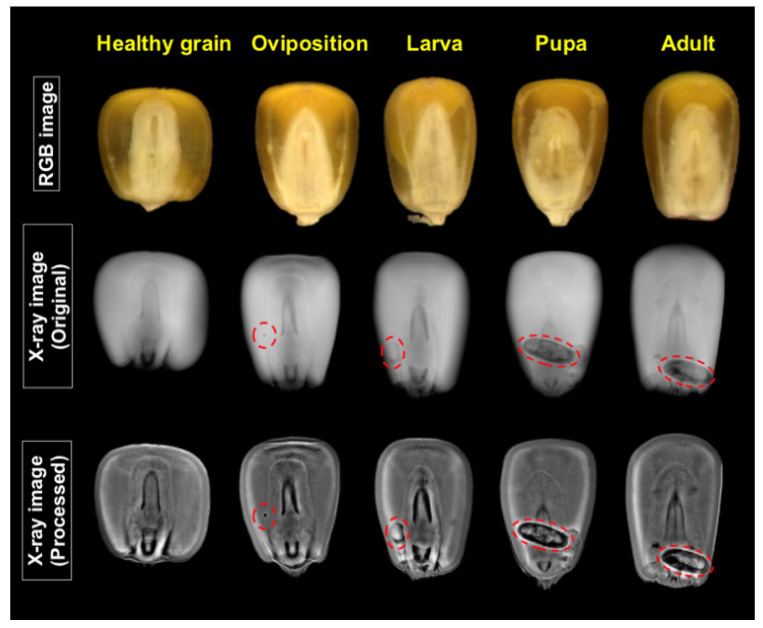
RGB images of non-infested maize grains (healthy grain) and grains infested with *Sitophilus zeamais* during oviposition, larva, pupa and adult stages and corresponding traditional X-ray images and after processing using peripheral equalization and calcification emphasis algorithms. Red circles/ellipses indicate infested regions inside the grain.

**Figure 4:**
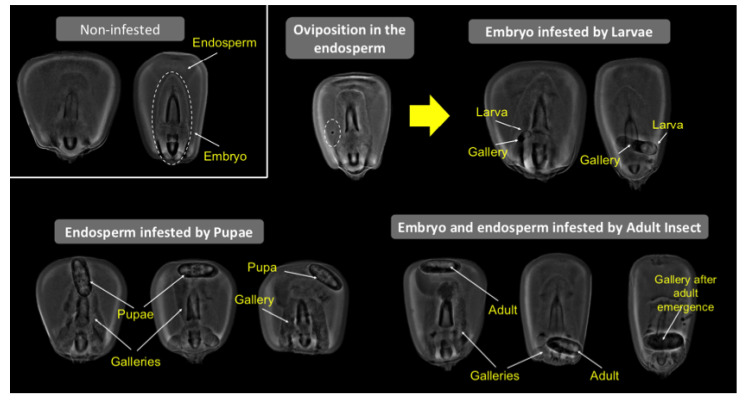
X-ray imagens processed by peripheral equalization and calcification emphasis algorithms for classes of non-infested maize grains and grains infested by *Sitophilus zeamais* showing damages (“galleries”) in different parts of the grain.

Throughout the infestation period (42 days), the RGB images did not show any alteration on the surface of the grains caused by *Sitophilus zeamais* (Figure 3). However, the radiographic images allowed the detection of the maize weevil in the internal parts of the grains at different developmental stages (oviposition, larva, pupa and adult) (Figure 3 and Figure 4). Nevertheless, without the application of the image pre-processing technique, it was difficult to detect the insect during the initial infestation, i.e., when the grains were infested with eggs (oviposition) or larvae (Figure 3). Meanwhile, the peripheral equalization and calcification emphasis algorithms improved the detection of *Sitophilus zeamais*, regardless of its stage of development (Figure 3 and Figure 4). In addition, the damages (“galleries”) caused by the insect were effectively detected in the grain (Figure 4).

### 3.2. Training, Validation, and Test Sets

**Figure 5:**
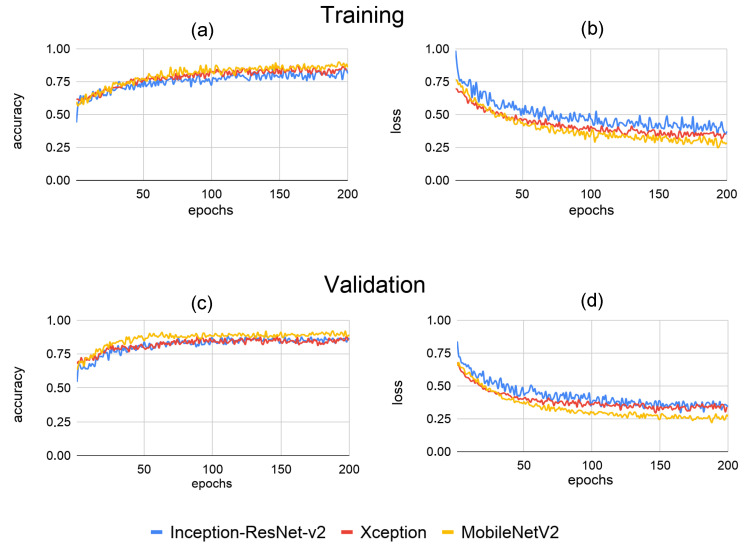
Performances on training (**a**,**b**) and validation (**c**,**d**) datasets during the learning process of Inception-ResNet-v2, Xception and MobileNetV2 architectures for classification of X-ray images from non-infested maize grains and grains infested with *Sitophilus zeamais*.

The models were trained for 200 epochs. During the learning process, the curves for each model indicated a predictive power for the classification of X-ray images from maize grains infested and not infested with *Sitophilus zeamais* (Figure 5). MobileNetV2 model showed slightly greater accuracy compared to the Xception and InceptionResNet-v2 architectures on training and validation sets. In the last epoch validation, the models achieved accuracies of 0.8926, 0.8571 and 0.8392 for MobileNetV2, InceptionResNet-v2 and Xception, respectively. The final loss, i.e., the prediction error of MobileNetV2, Xception and InceptionResNet-v2 models were 0.2676, 0.3605 and 0.3432.

**Figure 6:**
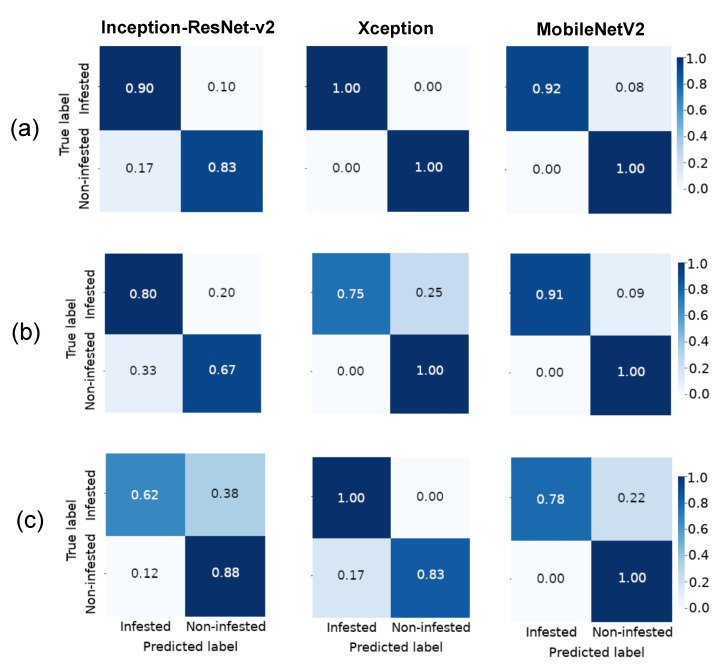
Confusion matrices for classification of X-ray images from non-infested maize grains and grains infested with *Sitophilus zeamais* based on Inception-ResNet-v2, Xception and MobileNetV2 classifier architectures on training (**a**), validation (**b**) and test (**c**) datasets.

The confusion matrices (Figure 6) of MobileNetV2 and Xception models achieved the highest hit rate for prediction of a non-infested class member on the validation set (1.00); however, the number of non-infested grains correctly classified based on the Xception model was reduced on the test set (0.83), while MobileNetV2 remained with the greatest performance (1.00). For class membership of infested grains, MobileNetV2 also showed the best performance (0.91) on the validation dataset. However, Xception had the highest hit rate on the test database (1.00).

**Figure 7:**
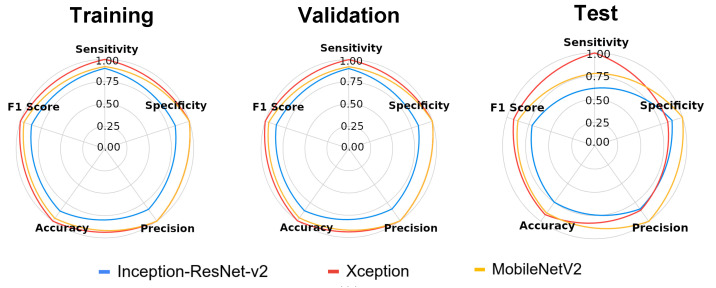
Star plot for the metrics of the Inception-ResNet-v2, Xcep-tion and MobileNetV2 architecture models tested for classification of radiographic images from maize grains infested and not infested with *Sitophilus zeamais*.

Classification performance in terms of precision, accuracy, sensitivity, specificity and F1 Score was also measured (Figure 7). The precision measures the number of X-ray images that were correctly classified as infested class (positive predictive value), with greater values shown for MobileNetV2 and Xception models on training and validation sets (1.00). In the test set, the precision of the Xception model was reduced to 0.85, while MobileNetV2 remained equal to 1.00. Again, both models achieved the best accuracies for training, validation and test sets (0.88–1.00).

The sensitivity of the Xception classifier had a superior performance mainly for the test dataset (1.00). Meanwhile, although higher specificity values were reached for Xception and MobileNetV2 on training and validation datasets, after learning the non-infested features on these datasets, the MobileNetV2 model showed the best specificity on the test set (1.00). This metric measures the number of non-infested grains that were correctly classified as non-infested. For F1 Score, the Xception model was more effective for all datasets (0.86–1.00) but with high performance also demonstrated for MobileNetV2 (0.88–0.96). F1 Score is a harmonic mean of precision and sensitivity when these metrics have the same weight, allowing the effectiveness of the model to be measured for the classification of infested grains.

## 4. Discussion

Maize grain plays an important role in the global economy, with increasing demand for food by population growth. Therefore, the development of rapid and accurate methods for monitoring the quality of grains has progressively increased. The maize weevil, *Sitophilus zeamais,* is a pest with high reproductive potential that can multiply rapidly during grain storage, especially without control of temperature and relative humidity [36]. The detection of the maize weevil is still a challenging task in the food industry even for trained analysts because the damage caused by weevils is not easily detected with the naked eye, except in the adult stage when punctures occur in the surface of the grain produced by the insect’s emergence.

X-ray imaging is a non-destructive technique to efficiently overcome such challenges, with rapid and reliable detection of weevils on the inner parts of a grain [8,37]. Maize grains infested by *Sitophilus zeamais* exhibited internal damages characterized by the absence of tissues that are referred to as “galleries”, and these regions appear darker on the radiographic images. This occurs due to the lower density of grain tissues [38] as a result of mass loss by insect feeding, while the electromagnetic radiation of X-rays is relatively short (high frequency), with wavelengths ranging from 0.01 to 10 nm [39]. The onset of insect attack occurs when the female, after mating, inserts the eggs in the grain, and then the eggs hatch into larvae, building galleries into the embryo or in the endosperm. Larvae, pupae and adults cause considerable damages by consuming the internal structures of the endosperm grain that do not exhibit any external symptoms (Figure 3).

Although the X-ray imaging technique provides several advantages in the detection of *Sitophilus zeamais*, there are still difficulties in detecting symptoms during the initial infestation period using unprocessed traditional X-ray images [40], as they usually show noise in the images, such as edges and contrast boundaries. In the current research, we tested contrast enhancement image processing based on peripheral equalization [24] and calcification emphasis algorithms [25], which are well-known in medicine to detect breast cancer. Since these image-processing techniques are automatic and display most of the information contained in the grayscale image, we expected to obtain a clearer identification of morphologic features of the insect and damages to the grain. Accordingly, our results showed that these algorithms enable a more accurate identification of insect attack, regardless of the grain location and insect growth (Figure 3 and Figure 4).

For image recognition, we proposed a method for classifying infested and non-infested maize grains based on CNN architectures, i.e., mathematical models that have been motivated by the functioning of the brain with the purpose to analyze data [11]. CNN architectures are composed of several layers of processing to learn representations of data with various levels of abstraction [12]. Therefore, we compared the performance of Inception-ResNet v2, Xception and MobileNetV2 architectures, which have improved the state of the art in object detection, segmentation and classification. The results showed that although the Inception-ResNet v2 model had a greater number of parameters (Table 1), the performance of MobileNetV2 and Xception models was superior as classifiers (Figure 5, Figure 6 and Figure 7). According to Chollet [30], the performance gains are not due to increased capacity but rather to a more efficient use of model parameters.

Modern deep neural network methods have revolutionized many areas of life science; however, their accuracy depends on advanced computing resources and the capacity of many mobile and embedded applications [31]. Despite this, the neural network architecture in the MobileNetV2 is suitable for mobile devices with limited resources, requiring no special operator, while maintaining accuracy. Hence, the new approach proposed based on X-ray images combined with MobileNetV2 architecture provides a markedly easier way to control *Sitophilus zeamais* infestation for safe human consumption of products with structural physical integrity.

The first successful application of CNNs as a deep learning application to images was shaped by LeCun, Bengio and Haffner in 1998 [41], but only in 2012 with the new architecture of CNN, AlexNet, did the statistical results for the image classification task really advance the state of the art [42]. Thereafter, progress on exploring computer vision has driven advances in the analysis of food images [43]. In this context, deep learning architectures such as MobileNetV2 combined with X-ray images can provide rapid predictions and recommendations for the next steps of the maize grain quality evaluation.

Currently, pest identification still depends on specialized technicians that may experience errors because the insect feeding damage does not exhibit external symptoms. High levels of insect debris cause odor and affect grain quality, making it unsuitable for food use [44]. Hence, rapid and accurate diagnosis of contaminated products allows for quick intervention. The method proposed does not require manual features; i.e., it can automatically learn how to recognize food contaminated with hidden storage pests, which makes it more reliable for grain inspection. Moreover, in the modern food industry, the application of computational techniques improves the speed of processing systems, reducing waste accumulation in the environment. However, the high cost of X-ray systems coupled with advanced image processing techniques may be a limitation for automatic inspection of *Sitophilus zeamais* infestation using enhanced radiographs of maize grains. Nevertheless, due to the rapid advancement in image analysis technologies and the growing demand for food, these tools may become more affordable in the near future.

## 5. Conclusions

The MobileNet and Xception architectures using enhanced radiographs are sensitive methods to distinguish non-infested grains from maize grain infested by *Sitophilus zeamais*, with overall accuracies higher than 0.88 for validation and test sets. The use of contrast enhancement techniques for X-ray images based on peripheral equalization and calcification emphasis algorithms improves the visualization of *Sitophilus zeamais* attack in such a way that it can be easily perceived during the initial infestation period. Therefore, the proposed deep learning approach provides an opportunity to make maize grain classification more predictable and efficient considering their internal patterns exhibited in X-ray images.

## Figures and Tables

**Table 1:** Comparison of convolutional neural network models in terms of parameters and accuracies in the ImageNet dataset.

	Inception-ResNet v2	Xception	MobileNetV2
Size	215 MB	88 MB	14 MB
Top-1 accuracy	0.803	0.790	0.713
Top-5 accuracy	0.953	0.945	0.901
Depth	572	126	88
Number of trainable parameters	1537	2049	1281
Number of non-trainable parameters	54,336,736	20,861,480	2,257,984
Total parameters	54,338,273	20,863,529	2,259,265

## Data Availability

The datasets generated for this study are available on request to the corresponding author.
